# Epidemiology of Thyroid Cancer: A Review of the National Cancer Database, 2000-2013

**DOI:** 10.7759/cureus.4127

**Published:** 2019-02-24

**Authors:** Evan Olson, Grant Wintheiser, Katrina M Wolfe, Jonathan Droessler, Peter T Silberstein

**Affiliations:** 1 Obstetrics and Gynecology, CHI Creighton University Medical Center, Omaha, USA; 2 Internal Medicine, CHI Creighton University Medical Center, Omaha, USA; 3 Physical Medicine and Rehabilitation, CHI Creighton University Medical Center, Omaha, USA; 4 Oncology, Creighton University School of Medicine, Omaha, USA

**Keywords:** thyroid cancer, stage iv thyroid cancer, overdiagnosis, epidemiology, incidence

## Abstract

Objective

To show the recent trends in thyroid cancer in the United States, elucidate the characteristics of stage IV thyroid cancer, and consider the effects of diagnostic testing on the rising incidence of thyroid cancer.

Design

A retrospective population-based study conducted using the National Cancer Database from 2000-2013 (NCDB). Demographics of patients presenting with stage IV thyroid cancer were compared to patients presenting with all other stages using the chi-square testing. The incidence rates were examined with the trend graphs.

Results

When compared to stages I-III, there was an increased incidence of stage IV thyroid cancer in: Medicare, lower level of education, lower income, advanced age, male sex, increased number of comorbidities, further distance from a treatment facility, and medullary/anaplastic histology. The incidence of thyroid cancer increased from 7.1 per 100,000 in 2000 to 17.6 per 100,000 in 2013. During this same time period, stage IV disease increased 1 per 100,000. The increase in the incidence of thyroid cancer was almost entirely due to an increase in papillary cancer.

Conclusions

The United States has continued to see a rise in the incidence of thyroid cancer over the last decade, largely due to the detection of papillary cancers. During this same time, the incidence of stage IV thyroid cancer increased as well. Because early diagnosis and treatment of an increasing number of potentially lethal cancers should lead to a decrease in metastatic disease, we suggest that the increasing incidence of thyroid cancer in the United States is due to overdiagnosis and that more aggressive disease is not being removed by early detection.

## Introduction

The incidence of thyroid cancer has rapidly increased in the United States (US) and other developed countries over the past 30 years [1-4]. A Surveillance, Epidemiology, and End Results Program (SEER)-based study found that from 1975 to 2009 there was a three-fold increase in incidence rates, from 4.9 to 14.3 per 100,000 individuals, while mortality rates stayed relatively constant at ~0.5 deaths per 100,000 [2]. This increase was primarily in small (<2 cm) papillary carcinomas, with an absolute increase in the rate of thyroid cancer in women four times greater than in men [2]. Based on prior studies analyzing the SEER program, there has been an annual rate increase in thyroid cancer of 3% in the US since the 1990s [3, 5].

Although some researchers believe this is a true increase in thyroid cancer [6-7], others believe an alternate theory: the increase is due to better diagnostic testing such as ultrasonography and fine-needle aspiration biopsy, resulting in the detection of disease that is unlikely to cause symptoms or death during a person’s lifetime [8-10]. Prior to the use of ultrasound for detecting thyroid cancer in the 1960s, thyroid cancer was commonly found in autopsies of individuals not previously diagnosed with the disease [3, 11]. Today, nearly 16% of computed tomography and magnetic resonance images in the US show incidental thyroid nodules, three-quarters of which are <15 mm. This suggests that many thyroid cancers may be indolent and non-lethal [10]. Thus, when treated with therapeutic interventions, there may be a limited benefit with the risk of potential harm. The reason for the increase in thyroid cancer continues to be debated, and many believe that both increased diagnostic scrutiny and true incidence play a role in this phenomenon [5, 12-14].

From the 1970s until the early 2000s there has been a marked change in patient demographics due to the declining proportion of anaplastic thyroid cancers (5.7% to 2.1%) and increase in papillary thyroid cancers (58% to 85.9%) – a trend towards increased incidence in women, those with access to health care and the wealthy [15]. However, due to relatively stable mortality rates, the indicators of poor outcome have remained: age >45 years, male sex, large tumor size, histology, advanced stage, extrathyroidal extension, lymphatic invasion, lack of radiation/surgical treatment and distant metastases [16-23]. The changing demographics of thyroid cancer demonstrated disparities in outcomes, and the paucity of studies in the current literature have led us to identify patient characteristics associated with an initial diagnosis of stage IV thyroid cancer and describe its recent incidence trend. This is the largest study to evaluate stage IV thyroid cancer.

## Materials and methods

Data sources and variables

This was a retrospective study that used the National Cancer Database (NCDB) to gather information on patients diagnosed with thyroid cancer between 2000 and 2013. The NCDB is a hospital-based cancer registry jointly sponsored by the American Cancer Society and the American College of Surgeons, which includes 70% of all cancers treated in the US [24].

The following characteristics were analyzed: race, insurance, education, income, age, sex, Charlson-Deyo Comorbidity Score, distance from the treatment facility, and histology. For characteristic analysis, the years 2000 to 2012 were analyzed.

The NCDB uses the Charlson-Deyo Comorbidity Score to represent the burden of comorbidity conditions. Educational data was recorded as an aggregate percentage of population without a high school degree residing in the patient’s zip code at the time of diagnosis. Data for education and income came from the 2012 US census and was determined based on patient zip codes. Patients within the “unknown” category for each characteristic were excluded from analysis.

Excess cases were calculated by subtracting the number of predicted cases of stage IV thyroid cancer from the actual number of cases. The predicted number of stage IV cases was determined assuming the null hypothesis that no subgroup influenced a number of stage IV cases – that within each category, the number of stage IV cases would be proportional to what percent that subcategory was in the population of all other stages within the database.

In gathering data to describe the trends in incidence, we restricted our analyses to the four major histological categories associated with thyroid cancer: papillary, follicular, medullary and anaplastic. The groups were presorted in the NCDB. Incidence rates for histological categories, stage IV and all thyroid cancers were examined for years 2000-2013. We adjusted the data to account for the NCDB capturing ~70% of incidence cancers in the US [24]. Because medullary and anaplastic thyroid cancers are rare and behave in a similar manner, they were combined into a single category labeled “poorly differentiated”.

When calculating the incidence of thyroid cancer in the US, we used historical data from the US Census Bureau to determine the US population for each year analyzed [25]. We divided the adjusted incidence of thyroid cancer each by the reported US population.

Statistical analysis

Patient characteristics were either ordinal or categorical, and they were presented as within-category percentages. The chi-square tests were used to calculate differences in the rate of demographic and clinical characteristics between patients initially diagnosed with stage IV thyroid cancer and all other stages. The level of statistical significance was set to P < 0.05. Trend graphs were generated. The data was presorted by the NCDB; therefore, we were unable to conduct any patient-level multivariable analysis.

## Results

Of the 343,386 patients with thyroid cancer reported to the NCDB between 2000 and 2012, 6.9% presented with stage IV upon diagnosis (Table 1). Patients with stage IV disease largely had private (46%) and Medicare insurance (40%). Most (71.4%) had a Charlson-Deyo Comorbidity Score of zero. They were predominately Caucasian (76.5%), female (56.4%) and elderly (57%; ≥ 60 years). Papillary carcinoma represented 49.4% of stage IV cases, followed by 33.7% medullary/anaplastic and 16.9% follicular (Table 2).

**Table 1:** Excess Number of Cases by Characteristic of Stage IV Thyroid Cancer Patients (2000-2012) *P < 0.001, ^Ɨ^2012 census data, ^∆ ^Excess cases were calculated by subtracting the number of predicted cases of stage IV thyroid cancer from the actual number of cases. The predicted number of stage IV cases was determined assuming the null hypothesis that no subgroup influenced the number of stage IV cases – that within each category, the number of stage IV cases would be proportional to what percent that subcategory was in the population of all of stage I-III cases.

		Stage IV (%)	Excess Cases^∆^ (#)
All patients			
	National Average	6.9	N/A
Race/Ethnicity			
	Caucasian	6.8	(-277)
	African American	6.9	1
	Hispanic	7.9	276
Insurance Status^*^			
	Not insured	8.7	181
	Medicaid	6.8	(-8)
	Medicare	14.6	5,034
	Private	4.6	(-5,150)
	Other government	6.1	(-57)
Population without a high school degree^*^^Ɨ^ (%)			
	≥23	8.4	589
	15-22.9	7.4	323
	11-14.9	7.1	156
	6-10.9	6.5	(-322)
	<6	5.9	(-746)
Median Income^*^^Ɨ^ ($)			
	<44,000	8.0	983
	44k -52,999	7.1	178
	53k - 68,999	6.7	(-162)
	≥69,000	5.9	(-997)
Age^*^ (years)			
	<50	2.5	(-7,579)
	50-59	7.7	648
	60-69	10.4	1,879
	≥70	18.9	5,052
Sex^*^			
	Male	12.1	4,464
	Female	5.2	(-4,464)
Charlson-Deyo Score^*^			
	0	6.8	(-1,557)
	1	10.3	1,053
	2+	14.2	504
Distance from treatment facility^*^ (mi)			
	<10	6.7	(-144)
	10-24	6.0	(-939)
	25-49	6.8	(-17)
	≥50	9.3	1,099
Histology^*^			
	Papillary	5.8	(-2,259)
	Follicular	3.9	(-2,979)
	Medullary/Anaplastic	20.1	5,237

**Table 2:** Epidemiology of Stage IV Thyroid Cancer Patients (2000-2012) ^Ɨ^ 2012 census data

		Stage IV (%)	All Stage (%)
Race/Ethnicity			
	Caucasian	76.5	77.8
	African American	7.2	7.2
	Hispanic	8.9	7.7
Insurance Status			
	Not insured	3.5	2.8
	Medicaid	5.1	5.2
	Medicare	40	18.9
	Private	46	68.2
	Other government	3.3	2.6
Population without a high school degree^ Ɨ^ (%)			
	≥23	13.6	11.2
	15-22.9	19.4	18.1
	11-14.9	16.7	16.1
	6-10.9	28.8	30.2
	<6	18.9	22.1
Median Income^ Ɨ^($)			
	<44,000	29.3	25.2
	44k -52,999	18.8	18.1
	53k - 68,999	23.3	24
	≥69,000	26	30.3
Age (years)			
	<50	17.8	50
	50-59	25.1	22.3
	60-69	23.4	15.4
	≥70	33.6	12.2
Sex			
	Male	43.6	24.7
	Female	56.4	75.3
Charlson-Deyo Score			
	0	71.4	72.5
	1	16	10.7
	2+	4.4	2.1
Distance from treatment facility (mi)			
	<10	35	35.8
	10-24	28.5	32.6
	25-49	15.8	15.9
	≥50	18	13.3
Histology			
	Papillary	49.4	59
	Follicular	16.9	29.5
	Medullary/Anaplastic	33.7	11.6

When compared to stages I-III, there was a greater increase of stage IV thyroid cancer in: Medicare, low high school graduation rates, annual median household income < $44,000, ≥ 60 years old, male sex, increased comorbidities and further distance from a treatment facility (Table 1, P < .05). Ethnicity/race had little association with the incidence of stage IV disease. Stage IV cancer incidence was higher in males (12.1%) compared to females (5.2%), and patients with Medicare insurance (14.6%) or who were uninsured (8.7%) were more likely to have stage IV thyroid cancer than those with private insurance (4.6%). Patients with 2+ co-morbidities were more than twice as likely to have stage IV thyroid cancer as those without co-morbidities (14.2% vs. 6.8%). Medullary/anaplastic cancers (20.1%) were much more likely to be stage IV than papillary (5.8%) or follicular cancers (3.9%, P < 0.05).

**Figure 1:**
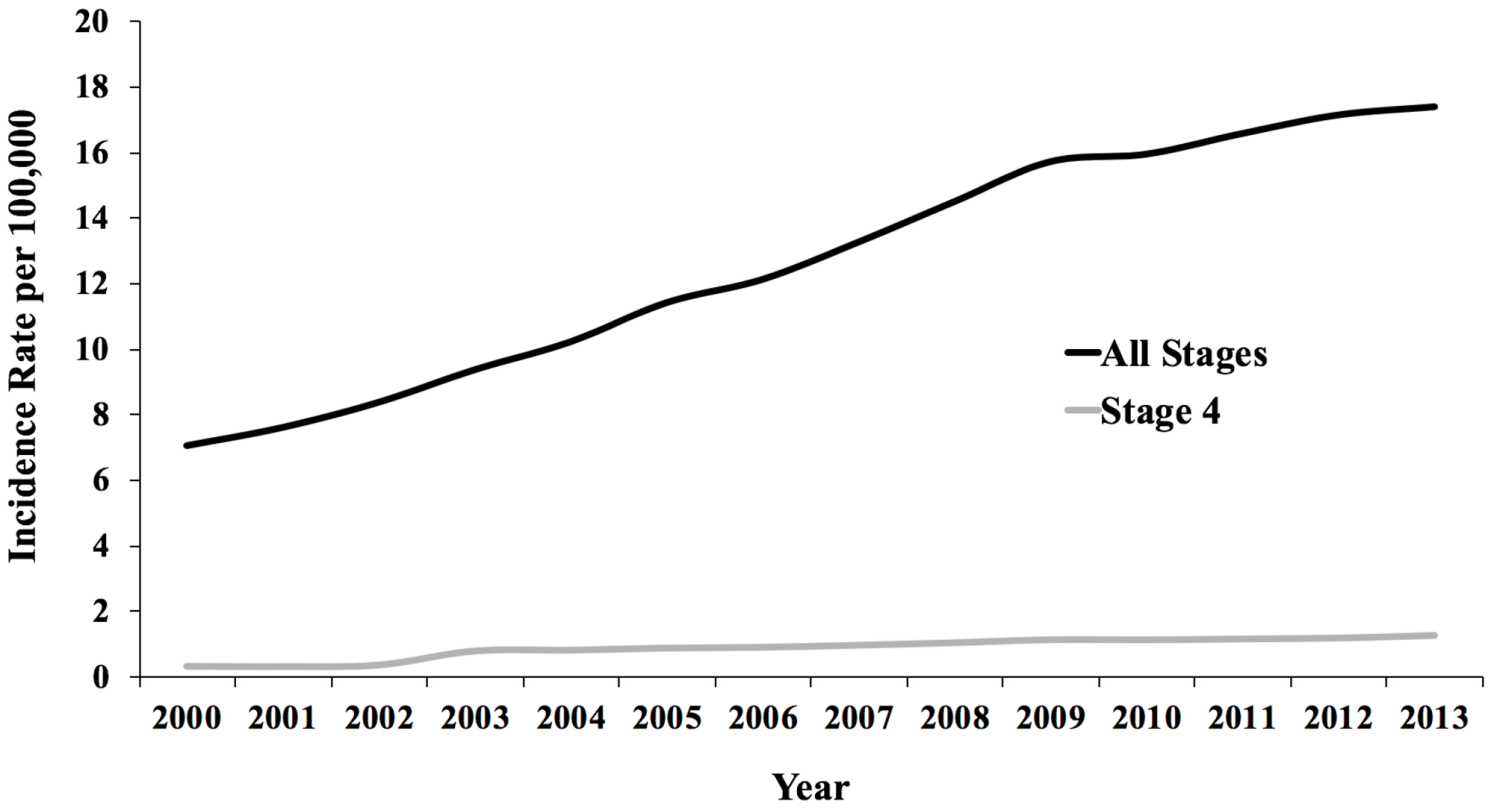
Trends in Incidence of Thyroid Cancer (2000-2013) in the United States

**Figure 2:**
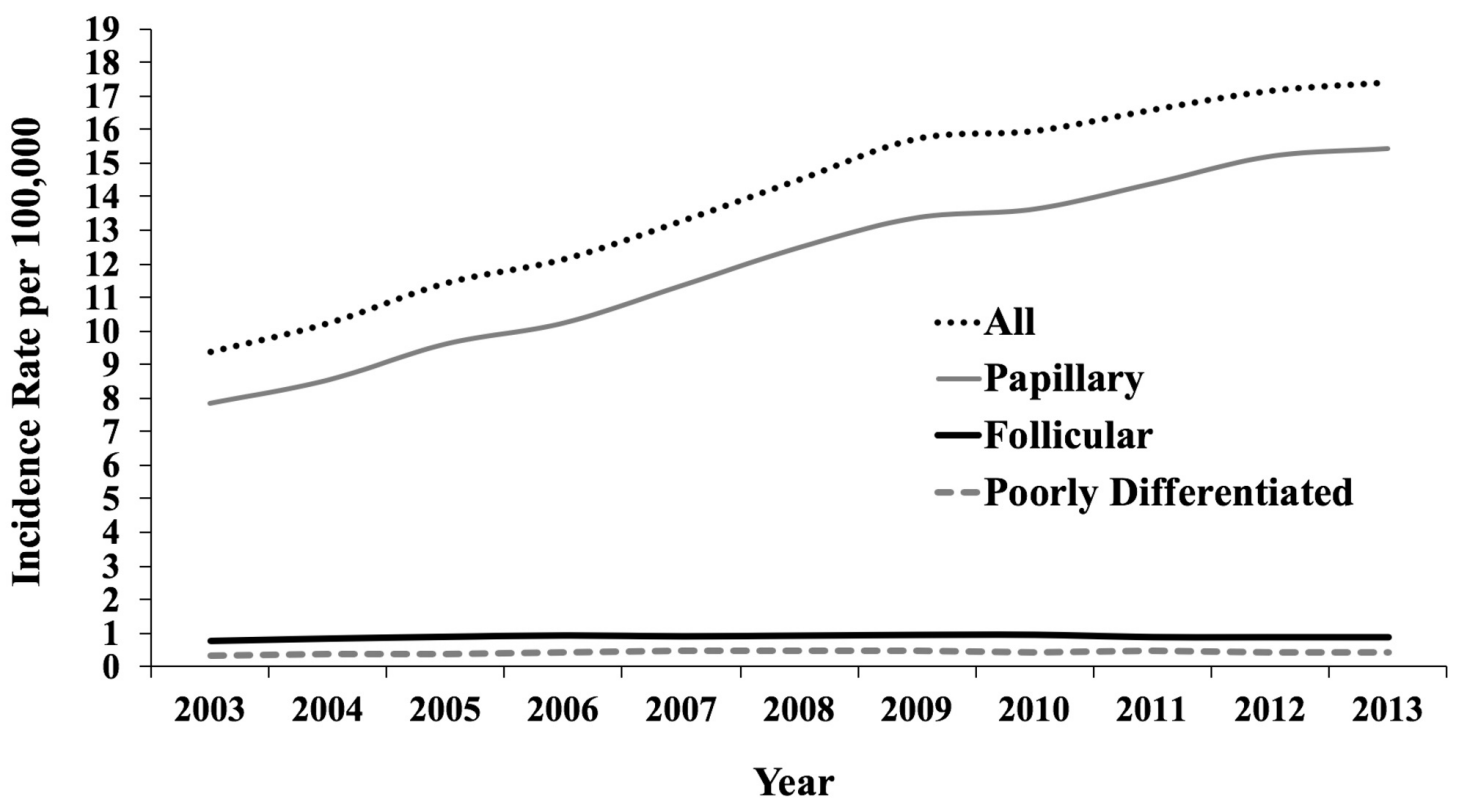
Trends in Incidence of Thyroid Cancer (2003-2013) in the United States by Histology

As shown in Figure 1 and Figure 2, the incidence of thyroid cancer increased from 7.1 per 100,000 in 2000 to 17.4 per 100,000 in 2013 – a 2.5-fold increase. During this same time period, stage IV thyroid cancer increased by 1.0 per 100,000 (Figure 1). The 10.3 per 100,000 increase in the incidence of thyroid cancer was almost entirely due to an increase in papillary cancer, which increased from 7.8 per 100,000 in 2003 to 15.4 per 100,000 in 2013 (Figure 2). Follicular and poorly differentiated cancers both minimally increased by 0.1 per 100,000 from 2003 to 2013.

## Discussion

Over the past 30 years, developed countries have seen a large increase in the incidence of thyroid cancer [1-4]. This “epidemic”, as some have termed it, is unique in that despite the increase in incidence mortality has remained relatively stable [2]. After all, early detection of cancer would presumably decrease mortality, which has not been observed. A few articles suggest that the increased incidence may be due to overdiagnosis: the diagnosis of cancers that if left untreated are unlikely to cause symptoms or death during a person’s lifetime [8-10].

Other researchers suggest that the increased incidence is due to factors beyond screening, such as access to health care and environmental, dietary and genetic influences [12-14]. The topic remains unsolved. Nonetheless, the demographics and clinical characteristics of thyroid cancer patients have been changing over the past decades [14] while mortality risk factors have remained the same [16-23]. The purpose of this paper was to elucidate the demographic and clinical characteristics of patients with stage IV thyroid cancer and show the newest epidemiologic data on thyroid cancer using the NCDB.

Our study demonstrated that certain characteristics were associated with a greater than predicted incidence of stage IV thyroid cancer compared to all other stages (Table 1). The largest difference between those with stage IV cancer and those with all other stages, >15% initially presenting with stage IV thyroid cancer, was seen with advanced age (over 60 years) and medullary/anaplastic cancer. Male sex, Medicare, 1+ Charlson-Deyo Comorbidity Score showed a medium size difference with 10-15% initially presenting with stage IV disease. Low education, low income, no insurance and a far distance from one’s treatment facility showed the smallest increased incidence difference, 8-10% initially presenting with stage IV disease. There was minimal difference in incidence by race/ethnicity in stage IV versus stages I-III. Our results are consistent with the previous research showing that males are more likely than females to present with advanced disease [16] and that the accelerating incidence in thyroid cancer is similar among ethnic groups [26].

We found that the incidence of thyroid cancer in the United States more than doubled from 2000 to 2013 and that most of the increase was due to papillary cancer. Similar findings have been demonstrated in prior papers [2-3]. Davies and Welch used the SEER program and data on thyroid cancer mortality from the National Vital Statistics System to show that the mortality rate from thyroid cancer was stable between 1975 and 2009 despite the rapid increase in incidence [2]. Our study demonstrates a similar finding that from 2000 to 2013 there was a 1 per 100,000 increase in the incidence rate of stage IV thyroid cancer while the total incidence of thyroid cancer increased over 10.3 per 100,000. The SEER data reports a total increase in thyroid cancer during this period to be 7.5 per 100,000. They also report the incidence of thyroid cancer in 2013 to be 15.1 per 100,000 compared to our finding of 17.4 per 100,000 [27]. The SEER program and the American Cancer Society both estimate approximately 64,000 new cases of thyroid cancer this year, which would yield an incidence of 19.7 per 100,000 individuals [27-28], an estimate that fits with our data.

Davies and Welch also showed, using the SEER program and data, that the rates of follicular, medullary and anaplastic thyroid cancers show no significant change from 1973 to 2002 (P>.20 for trend) [3]. Our study found that this trend of stability was maintained from 2003 to 2013: 0.1 per 100,000 increase for follicular and medullary/anaplastic thyroid cancers. Due to their finding of an increased incidence of papillary cancers, despite stable mortality and incidence of aggressive thyroid cancers, Davies and Welch attributed the rise in thyroid cancer to overdiagnosis [3].

Other countries have seen similar increases in thyroid cancer. From 1993 to 2011, South Korea witnessed a 15-fold increase in thyroid cancer with nearly the entire increase attributed to papillary cancers, yet they failed to observe a decrease in mortality [29]. These numbers far outpace those we report in our study in the US, but they align with our findings of a rapid increase in papillary thyroid cancer. Ahn, Kim and Welch show that in South Korea the incidence of thyroid cancer increased from 10 to 70 per 100,000 individuals from 2000 to 2011 during which we saw the incidence increase from 7.1 to 16.6 per 100,000 individuals in the US. Of those being diagnosed in South Korea, approximately two-thirds undergo radical thyroidectomy and one-third undergo subtotal thyroidectomy. Beyond surgical complications such as vocal cord paralysis, these possibly avoidable thyroidectomies leave many patients dependent upon life-long thyroid-replacement. Ahn, Kim and Welch urge other countries to examine their practices of thyroid cancer screening to prevent the “epidemic” that has hit South Korea [29].

The current study does not provide all the answers, but the evidence presented in this paper shows that there has been a marked increase in the diagnosis of thyroid cancer without a reduction of deadly thyroid cancer. This suggests overdiagnosis. If we want to avoid unnecessary surgeries with life-long consequences, we may need to re-evaluate how we diagnose and treat thyroid cancer.

Our study has certain limitations, which include retrospective study design, utilization of a large secondary database with a potential for miscoding, missing data and lack of patient-level data for multivariable analysis. The differences between all of the groups are statistically significant, but the actual difference is small in some subgroups, as reviewed in the paper, and may have minimal clinical significance. Such results are likely due to our large sample size. The NCDB does not include data from non-Commission on Cancer-approved hospitals, which are typically smaller, located in rural settings, and have less cancer-related services available to patients.

Prior studies have evaluated the indicators of poor outcome on thyroid cancer, but only a few studies have specifically looked at stage IV thyroid cancer and its patient characteristics. To the best of the authors’ knowledge, our study is one of the largest to evaluate stage thyroid cancer and first to specifically evaluate stage IV disease with the NCDB.

## Conclusions

The following characteristics are seen at a higher than predicted incidence in stage IV thyroid cancer, in order from largest to smallest increased incidence: medullary or anaplastic thyroid cancer, advanced age, Medicare, increased number of comorbidities, male sex, far distance from a treatment facility, no insurance, lower level of education and lower income. The United States has continued to see a rise in the incidence of thyroid cancer over the last decade which has predominately been due to the detection of papillary cancers. Furthermore, the incidence of stage IV thyroid cancer did not decrease from 2000 to 2013. Because early diagnosis and treatment of an increasing number of potentially lethal cancers should decrease metastatic disease, we suggest that the increasing incidence of thyroid cancer in the US is due to overdiagnosis and that more aggressive disease is not being removed with early detection
